# Persistent homology in graph power filtrations

**DOI:** 10.1098/rsos.160228

**Published:** 2016-10-26

**Authors:** Allen D. Parks, David J. Marchette

**Affiliations:** Electromagnetic and Sensor Systems Department, Naval Surface Warfare Center Dahlgren Division, 18444 Frontage Road Suite 327, Dahlgren, VA 22448-5161, USA

**Keywords:** homology, persistence, graph topology, graph power

## Abstract

The persistence of homological features in simplicial complex representations of big datasets in *R^*n*^* resulting from Vietoris–Rips or Čech filtrations is commonly used to probe the topological structure of such datasets. In this paper, the notion of homological persistence in simplicial complexes obtained from power filtrations of graphs is introduced. Specifically, the *r*th complex, *r* ≥ 1, in such a power filtration is the clique complex of the *r*th power *G*^*r*^ of a simple graph *G*. Because the graph distance in *G* is the relevant proximity parameter, unlike a Euclidean filtration of a dataset where regional scale differences can be an issue, persistence in power filtrations provides a scale-free insight into the topology of *G*. It is shown that for a power filtration of *G*, the girth of *G* defines an *r* range over which the homology of the complexes in the filtration are guaranteed to persist in all dimensions. The role of chordal graphs as trivial homology delimiters in power filtrations is also discussed and the related notions of ‘persistent triviality’, ‘transient noise’ and ‘persistent periodicity’ in power filtrations are introduced.

## 1. Introduction

Topological data analysis is concerned with determining the topological structure of data [1]. One such approach to analysing large sets of discrete data points in $R^{n}$ is to convert the dataset into a global topological object by replacing the dataset with a simplicial complex indexed by a Euclidean distance proximity parameter that defines the simplices of the complex. The powerful mathematical machinery of algebraic topology, e.g. [2], can then be applied to the complex in order to understand the fundamental topological properties of the dataset in terms of the topologically invariant homology groups associated with the data's simplicial complex representation. When combined with persistence theory and barcode theory, e.g. [3], these homology groups can often provide valuable insights about the underlying phenomena represented by the data. These methods have been used to study such diverse areas as sensor network coverage [4], random graphs and complex networks [5], shape analysis [6], brain topology [7], the evolution of viruses [8] and ancestral genetic history [9]. The intrinsic mathematical appeal of these methods has also prompted mathematicians to further formalize their description and extend their utility (e.g. [10,11]).

Traditional persistence theory typically uses Vietoris–Rips (Rips for short) or Čech Euclidean filtrations of $R^{n}$ datasets to generate a series of Rips or Čech simplicial complexes, each associated with a different value of the varying Euclidean distance proximity parameter. This paper introduces a variant to this persistence approach to topological analysis that is based upon the *power filtration* of a simple graph *G*. It uses the graph distance r≥1 in *G* as the associated proximity parameter and generates the filtration by increasing the value of *r*. The *r*th complex in the filtration is the clique complex of the *r*th power $G^{r}$ of *G* (such a filtration is called an *r filtration* and each complex in the filtration is called an *r complex*: note that an *r* complex is a Rips complex whose simplices are subsets of the vertices of *G* that are a distance of at most *r* from each other in the discrete metric space defined by *G*). Although persistent homology in a Euclidean filtration yields information about a dataset's topology in $R^{n}$ from the perspective of a one-parameter family of complexes whose vertices are the data points and whose simplices are defined by varying the Euclidean distance proximity parameter in $R^{n}$, persistent homology in a power filtration of a graph provides topological insights about the graph from complexes whose vertices are those of the graph and whose simplices are defined using graph distance within the graph as the variable proximity parameter. Thus, unlike Euclidean filtrations where regional scale differences can be an issue, power filtrations can provide scale-free insights into graph topology. In both Euclidean and *r* filtrations, persistent homology features are considered to reflect important topological properties. Homology features which do not persist are generally regarded as relatively unimportant ‘topological noise’.

Because of the increasing interest in topological data analysis, much recent attention has been devoted to the development of software packages which can perform persistent homology computations with relative efficiency, e.g. [12–14] (it is interesting to note that a quantum mechanical algorithm has recently been developed that will exponentially speed up these computations—but alas—the quantum computers required to execute the algorithm do not yet exist [15]). It is shown below that in certain cases the girth of *G* can be used to reduce or eliminate such computations for *r* filtrations of *G* by defining not only a range Δr of *r* index values for which all of the homology features in all dimensions of the associated *r* complexes remain unchanged, but also a lower bound for the persistence lifetimes of every such feature. Although quantifying the girth of *G* can also place demands on computational resources, its evaluation can be cost-effective when compared with the resources required to compute all of the homology groups for each *r* complex in the Δr range (as noted below—the easily computed Randić index can be used to bound girth).

It will also be shown that chordal graphs—which have homologically trivial clique complexes—serve several important functions in *r* filtrations. As the power filtration of a connected graph *G stabilizes* at some power *r_s_*(*G*) (or *r_s_* when the graph referenced is clear) as a complete graph (which is a chordal graph), the associated filtration of *r* complexes stabilizes at the *stabilization distance r_s_* (*G*) as a homologically trivial simplex. In addition, if—in a power filtration of a connected graph *G* – $G^{r_{c}(G)}\equiv G^{r_{c}}$, $1<r_{c}<r_{s}$, is a chordal graph, then—until the stabilization distance $r_{s}$ is reached—the complexes associated with ${\left(G^{r_{c}}\right)}^{2j+1},j\geq 1$, are all homologically trivial. This is a *persistent periodic homology* feature peculiar to *r* filtrations that can also induce persistent periodicity in *transient noise*, i.e. ‘topological noise’ having the smallest possible lifetime in an *r* filtration. It can also be the case that all of the complexes associated with ${\left(G^{r_{c}}\right)}^{j},j\geq 1$, are homologically trivial (when $G^{r_{c}}$ contains no sunflower subgraph—see the next section). If this occurs the filtration exhibits *persistent trivial homology* when $r\geq r_{c}$. For the general extreme case where the complex associated with $G^{r}$ is homologically trivial for $1\leq r\leq r_{S}$, then the filtration is said to exhibit *persistent triviality*.

In order to make this paper relatively self-contained, relevant definitions and terminology are summarized in the next section. The theory of persistent homology in *r* filtrations (i.e. *r persistence*) is introduced in §3. Required preliminary lemmas are stated in §4 and the main results are developed in §5. Illustrative examples are presented in §6 and closing remarks comprise the paper's final section.

## 2. Definitions and terminology

A simple *graph G* is a pair (V(G),E(G)), where V(G) is a finite non-empty set of *vertices* and E(G) is either a set of doubleton subsets of V(G) called *edges* or the empty set. The *order* of *G* is the cardinality |V(G)| of its vertex set and the *size* of *G* is the cardinality |E(G)| of its edge set. Two vertices u,v∈V(G) are *adjacent vertices* when e={u,v}∈E(G), in which case *e* is said to *join u* and *v* while *u* and *e* and *v* and *e* are said to be *incident*. Two distinct edges in *G* are *adjacent edges* if they have a common vertex. The *degree* $\mathrm{de}g_{G}(u)$ of vertex u∈V(G) is the number of edges incident to *u* and the sum $\sum_{\left\{u,v\}\in E(G\right)}{\left[\deg(u)\deg(v)\right]}^{-1/2}$ is the *Randić index* R(G) of *G*. A graph is *regular* if each of its vertices has the same degree. Graphs *G* and *F* are *isomorphic graphs* (denoted ${\;}^{\prime }G≅F^{\prime }$) if there is a bijective map θ:V(G)→V(F) such that {θ(u),θ(v)}∈E(F) when {u,v}∈E(G).

A u−v *walk* is an alternating sequence of vertices and edges beginning with *u* and ending with *v* such that every edge joins the vertices immediately preceding and following it. A u−v *path* is a u−v walk in which no vertex is repeated and the number of edges it contains is its *length*. In this case, *u* is said to be connected to *v*. *G* is *connected* if its order is one or if every two vertices in *G* are connected. The *graph distance* d(u,v) between u,v∈V(G) is the minimum length of all u−v paths and the diameter diam(G) of *G* is $\mathrm{ma}x_{u,v\in V(G)}d(u,v)$. Clearly, $r_{s}(G)=\mathrm{diam}(G)$. A u−v path for which u=v and which contains at least three edges is a *cycle*. The graph $C_{n}$ is the *cycle graph* on *n* vertices. The *length* of a cycle is the number of edges contained within it and the shortest cycle of *G* is the *girth* g(G) of *G*. A *chord* of a cycle is an edge between non-consecutive vertices in the cycle.

The *r*th *power* $G^{r},r\geq 1$, of *G* is the graph with vertex set $V(G^{r})=V(G)$ and for which $\left\{u,v\}\in E(G^{r}\right)$ if, and only if, the distance between *u* and *v* in *G* is at most *r*. A graph is a *chordal graph* if every cycle of length of at least four has a chord. A graph $K_{n}$ is a *complete graph* on *n* vertices when n=|V(G)|=1 or every two of its vertices are adjacent. A graph *F* is a *subgraph* of *G,* denoted F⊆G, if V(F)⊆V(G) and E(F)⊆E(G). If U(G)⊂V(G), then the subgraph of *G* induced by U(G) is the graph (U(G),E(G)), where {u,v}∈E(G)⊂E(G) if, and only if, {u,v}⊂U(G). A cycle $C_{n}$ in *G* is a *chordal cycle* if the subgraph induced by $C_{n}$ is chordal. A *sunflower* is the graph $S_{n},n\geq 3$, consisting of a chordal cycle $C_{n}=(v_{1},v_{2},\ldots ,v_{n})$ and *n* independent vertices $\left\{u_{1},u_{2},\ldots ,u_{n}\right\}$ such that for i∈{1,2,…,n}, $u_{i}$ is adjacent to only $v_{i}$ and $v_{j},j=i-1\;\mathrm{mod}\;n$. $S_{n}$ is *suspended* if there is a vertex $w\notin S_{n}$ that is adjacent to at least one vertex pair $u_{j},u_{k},j\neq k\pm 1\;\mathrm{mod}\;n$. If $G_{1},G_{2},\ldots ,G_{k}$ are not induced subgraphs of *G*, then *G* is $\left(G_{1},G_{2},\ldots ,G_{n}\right)$-*free*. A *clique* in *G* is either a vertex or a complete subgraph of *G*.

An *abstract simplicial complex S* on a finite set *A* is a family of sets {σ∈S:σ⊆A} such that: (i) {a}∈S for all a∈A and (ii) if σ∈S, then so is every subset of *σ*. The elements of *A* are the *vertices* $S^{\left(0\right)}$ *of S* and each *k*-simplex $\sigma =\{a_{0},a_{1},\ldots ,a_{k}\}$ is a *k*-dimensional *face of S*. The *clique complex* Cℓ(G) of a graph *G* is the abstract simplicial complex whose faces are the cliques of *G*. Associated with each complex *S* is a chain complex of abelian groups $\Gamma_{k}(S)$ and homomorphisms $\partial_{k+1}:\Gamma_{k+1}(S)\to \Gamma_{k}(S),k\geq 0,$ where $\ker\;\partial_{k}$ are the *k*-cycles in *S* and $im\partial_{k+1}$ are the *k*-boundaries in *S*. If $\rho_{k}(S)$ is the number of *k*-simplices in *S*, then $\Gamma_{k}(S)$ is isomorphic to (denoted ‘≈‘) the direct sum ‘⊕’ of $\rho_{k}(S)$ copies of the additive group of integers Z. The *k*th *homology group* of *S* is the quotient group $H_{k}(S)=\ker\;\partial_{k}/\mathrm{im}\partial_{k+1}$ which captures equivalence classes of non-bounding *k*-cycles by factoring out boundary cycles. If *S* is comprised of *m* connected components, then $H_{0}(S)\approx Z⊕Z⊕\cdots ⊕Z$ (*m* copies of Z) and if *S* is of dimension *δ*, then it has δ+1 homology groups. *S* is *homologically trivial* when $H_{0}(S)\approx Z$ and $H_{k}(S)\approx 0,k\geq 1$.

If *S* and *T* are abstract simplicial complexes, then a *simplicial map* φ:S→T is a map $\varphi :S^{\left(0\right)}\to T^{\left(0\right)}$ such that whenever $\left\{a_{0},a_{1},\ldots ,a_{k}\right\}$ is a simplex in *S*, then $\left\{\varphi (a_{0}),\varphi (a_{1}),\ldots ,\varphi (a_{k})\right\}$ is a simplex in *T*. The simplicial map φ:S→T *induces homomorphisms* $\varphi_{*}:H_{k}(S)\to H_{k}(T)$ between the homology groups of *S* and *T*. Complexes *S* and *T* are *homotopy equivalent* (denoted ${\;}^{\prime }S∼T^{\prime }$) if there are maps f:S→T and h:T→S such that $h\circ f≃i_{S}$ and $f\circ h≃i_{T}$ (here ‘∘’ denotes ‘composition of maps’, ‘≃’ denotes ‘homotopy of maps’, and $i_{S(T)}$ is the identity map on S(T)). If *S* and *T* are homotopy equivalent, then $H_{k}(S)\approx H_{k}(T),k\;\geq 0$.

## 3. Persistent homology theory for power filtrations of graphs

When applied to a complex, homology detects the presence of connected components, holes and voids in the complex. Rather than use the homology of a single complex as a description of the topology of a graph, it can be preferable to describe a graph's topology by identifying topological features detected by the homology that persist in a filtration of the graph. As already mentioned, persistent homological features can indicate potentially important topological properties of a graph, whereas features which do not persist can generally be regarded as relatively unimportant ‘topological noise’.

Useful insights into the topology of a simple graph *G* can be obtained from an understanding of the homology of clique complexes derived from the *r*th powers $G^{r}$ of *G* (e.g. if *G* is connected and diam(G) is known, *r* can provide a measure of how close *G* is to being a homologically trivial simplex). To this end, let *r* vary over an appropriate distance range 1≤r≤p within *G* to produce the *r* filtration and induced homology sequence given by the diagrams

3.1 $$C\ell (G)\overset{i_{1}}{⟶}C\ell (G^{2})\overset{i_{2}}{⟶}\cdots \overset{i_{p-1}}{⟶}C\ell (G^{p})$$

and

3.2 $$H_{k}(C\ell (G))\overset{\varphi_{*1}}{⟶}H_{k}(C\ell (G^{2}))\overset{\varphi_{*2}}{⟶}\cdots \overset{\varphi_{*p-1}}{⟶}H_{k}(C\ell (G^{p})),\;k\geq 0,$$

where ‘$\overset{i_{j}}{\to }$’ and ‘$\overset{\varphi_{*j}}{\to }$’ denote simplicial inclusion maps and group homomorphisms, respectively. As already noted in §1, if *G* is connected, then the filtration stabilizes with the homologically trivial simplex $C\ell (G^{r_{s}})$. In this case, the homology sequence for k>0 can be extended to include the groups

3.3 $$H_{k}(C\ell (G^{p}))\overset{\varphi_{*p}}{⟶}\cdots \overset{\varphi_{*r_{s}-2}}{⟶}H_{k}(C\ell (G^{r_{s}-1}))\overset{\varphi_{*r_{s}-1}}{⟶}H_{k}(C\ell (G^{r_{s}}))\approx 0,\;k>0,$$

where $\varphi_{*r_{s}-1}$ is obviously a zero homomorphism.

A non-zero homology class $[c]\in H_{k}(C\ell (G^{j})),k\geq 0$, represented by the cycle *c* is ‘born’ at $C\ell (G^{j})$ if [*c*] is not in the image of $\varphi_{*t}$ for t<j. It ‘dies’ at $C\ell (G^{l})$ if l>j is the smallest integer such that the image of [*c*] is 0 in $H_{k}(C\ell (G^{l}))$, i.e. if

3.4 $$\psi ([c])\equiv \varphi_{*l-2}\circ \cdots \circ \varphi_{*j}([c])\neq 0,$$

but

3.5 $$\varphi_{*l-1}\circ \psi ([c])=0,$$

where ‘∘’ denotes ‘composition of homomorphisms’. The *r persistence lifetime of c* is then λ(c)=l−j and is represented by the lifetime interval [j,l]. A non-zero homology class [*c*] that is ‘born’ at Cℓ(G) and ‘dies’ at $C\ell (G^{r_{s}})$ has a lifetime $\lambda (c)=r_{s}(G)-1$ and is said to be an $r_{s}(G)$ *survivor*. *Transient noise* is a class $0\neq [c]\in H_{k}(C\ell (G^{j})),k>0$, such that [*c*] is not in the image of $\varphi_{*j-1}$ but $\varphi_{*j}([c])=0,$ i.e. it is ‘born’ at $C\ell (G^{j})$ and ‘dies’ at $C\ell (G^{j+1})$. The lifetime of transient noise is λ(c)=1 and is represented by the lifetime interval [j,j+1]. Note that transient noise corresponds to the smallest possible non-zero lifetime that can exist in an *r* filtration.

A visualization of a complete *r* persistence analysis of *G* is given by the *complete r persistence barcode for G* which is a graphical representation of the multiset of all lifetime intervals for finite *k*. The *binary r persistence barcode for G* is a binary string β(G) of length $r_{s}(G)$ where the *r*th entry corresponds to $C\ell (G^{r})$ and is 0(1) if $H_{k}(C\ell (G^{r}))\approx 0,k>0\;(≉0$ for some k>0). For example, if β(G) is a string of $r_{S}$ zeros, then *G* exhibits persistent triviality which indicates that the topology of *G* is effectively homologically featureless (and possibly uninteresting) for all powers of *G*. However, if 010 is a substring of β(G), then for the *r* corresponding to the position of the 1 in β(G), the complex $C\ell (G^{r})$ generates transient noise in at least one dimension k>0. This indicates that relationships exist in subsets of *G*'s vertices that are manifested as short-lived (but possibly interesting) topological features in $C\ell (G^{r})$.

## 4. Preliminary lemmas

Results needed to prove or discuss the main results of this paper are presented in this section for the reader's convenience. The following lemmas have been established elsewhere and are stated here without proof.

**Lemma 4.1 ([16]).**

*If* g(G)≥3l+1,l>2, *then* $C\ell (G^{r-1})∼C\ell (G^{r})$ *when* 2≤r≤l.

**Lemma 4.2 ([17,18]).**

*If G is a connected graph of order at least 3 with Randić index* R(G) *and girth* g(G), *then* R(G)−(1/2)|V(G)|+3≤g(G)≤R(G)+(1/2)|V(G)|, *where equality applies on the left* (*right*) *if and only if G is a regular graph with a triangle* $\left(G\approx C_{\left|V(G)\right|}\right)$.

**Lemma 4.3 ([19]).**

*If G is a connected chordal graph, then* Cℓ(G) *is homologically trivial*.

**Lemma 4.4 ([20]).**

*If G is a chordal graph with no sunflower, then* $G^{r}$ *is chordal for all* r≥1.

**Lemma 4.5 ([21]).**

*If G is a chordal graph, then so is* $G^{2j+1}$ *for any* j≥1.

**Lemma 4.6 ([20]).**

*If G chordal and* $G^{2}$ *is not chordal, then G has at least one sunflower* $S_{n},n\geq 4$, *which is not suspended in G*.

## 5. Main results

In what follows, it is assumed that *G* is a simple graph. However, before continuing—for completeness—the above observations concerning *r* filtration stabilization are generalized as the following theorem. Since the consequence of the theorem is obvious, the theorem is stated without proof.

**Theorem 5.1.**

*If G comprises m connected components* $G_{1},G_{2},\ldots ,G_{m}$, *then* $r_{s}(G)=\mathrm{ma}x_{j=1,2,\ldots m}\;r_{s}(G_{j})$.

### 5.1. Persistent homology in *r* filtrations

The last theorem suggests the following persistent homology theorem for *r* filtrations:

**Theorem 5.2.**

*If G has* m≥1 *connected components*, *then the m non-zero homology classes of*

5.1 $$H_{0}(C\ell (G))\approx Z⊕Z⊕\cdots ⊕Z(m\;\mathrm{copies}\;\mathrm{of}\;Z),$$

*are* $r_{s}(G)$ *survivors*.

Proof.

This is an obvious consequence of theorem 5.1 and the fact that since *r* is a graph distance in *G* and the number of connected components remains invariant in $G^{r},r\geq 1$. ▪

Hereafter, for the sake of simplicity and without loss of generality, it will be assumed that *G* is a connected graph.

The next result shows that the girth of *G* defines a power index range over which non-zero homology classes of Cℓ(G) are guaranteed to persist.

**Theorem 5.3.**

*For some counting number* l>2, *let* g(G)≥3l+1*. If* $0\neq [c]\in H_{k}(C\ell (G)),k>0$, *then* λ(c)≥l.

Proof.

Lemma 4.1 implies that $C\ell (G^{r-1})$ and $C\ell (G^{r})$ are homotopy equivalent complexes for 2≤r≤l. Consequently, their homology groups are isomorphic for k>0 and the sequence

5.2 $$\begin{array}{r} H_{k}(C\ell (G))\overset{i_{*1}}{⟶}H_{k}(C\ell (G^{2}))\overset{i_{*2}}{⟶}\cdots \overset{i_{*l-1}}{⟶}H_{k}(C\ell (G^{l}))\overset{\varphi_{*l}}{⟶}H_{k}(C\ell (G^{l+1}))\overset{\varphi_{*l+1}}{⟶}\cdots \overset{\varphi_{*r_{s}-1}}{⟶}H_{k}(C\ell (G^{r_{s}}))\approx 0,k>0, \end{array}$$

of homology groups and induced homomorphisms exists, where each $i_{*j}$ is an isomorphism. If $0\neq [c]\in H_{k}(C\ell (G))$, then—since each $i_{*j}$ in the sequence is an isomorphism—it must be the case that

5.3 $$\Phi ([c])\equiv i_{*l-1}\circ i_{*l-2}\circ \cdots \circ i_{*1}([c])\neq 0.$$

Now let $q\in \{l,l+1,l+2,\ldots ,r_{s}-1\}$ be the smallest integer such that $\varphi_{*q}\circ \varphi_{*q-1}\circ \cdots \circ \varphi_{*l}\circ \Phi ([c])=0$, in which case λ(c)=q+1−1=q≥l. ▪

It is clear that theorem 5.3 only applies when g(G)≥10 and is most useful for graphs with extremely large girths. Lemma 4.2 provides a relatively straightforward and quick method for determining if g(G) is large enough to apply theorem 5.3.

The importance of chordal graphs as delimiters for persistent trivial homology in *r* filtrations is expressed in the next theorem. Chordal graphs have been studied extensively over the last several decades and efficient algorithms have been developed which can recognize when a graph is chordal (e.g. [22]).

**Theorem 5.4.**

*Suppose that* $G^{r_{c}}$ *is an* $\left(S_{n}\right)$*-free chordal graph. Then* $\lambda (c)\leq r_{c}-1$ *for every* $0\neq [c]\in H_{k}(C\ell (G^{r}))$, $k>0,r\;<\;r_{c}$.

Proof.

Since $G^{r_{c}}$ is a chordal graph with no sunflower, then ${\left(G^{r_{c}}\right)}^{j}$ is chordal for all j≥1 (lemma 4.4). Consequently, $C\ell ({\left(G^{r_{c}}\right)}^{j}),j\geq 1$, are homologically trivial complexes so that $H_{k}(C\ell ({\left(G^{r_{c}}\right)}^{j}))\approx 0,k>0$, for j≥1 (lemma 4.3). It must therefore be the case that for k>0, all non-zero homology classes [*c*] are ‘born’ and ‘die’ in $C\ell (G^{r})$ with $r<r_{c}$. Since r=1 is the smallest power index for which [c]≠0 can be ‘born’ and $r=r_{c}$ is the largest power index for which [c]≠0 can ‘die’, then the persistence lifetime of *c* can be no greater than $r_{c}-1$ when k>0. ▪

### 5.2. Persistent periodicity and transient noise in *r* filtrations

While it is the case that all chordal graphs are not closed under powers [23] (a situation for which closure occurs (lemma 4.4) has been applied in theorem 5.4), it is nonetheless true that every odd power of a chordal graph *G* is also chordal (lemma 4.5). Thus, the presence of a chordal graph at $r_{c}(G)<r_{s}(G)$ in an *r* filtration—at a minimum—guarantees that at least a *periodic homological triviality persists* in the associated complexes in the filtration. In what follows, it will be assumed that all power indices do not exceed the stabilization distance $r_{s}(G)$.

**Theorem 5.5.**

*If* $G^{r_{c}}$ *is a chordal graph, then* $H_{k}(C\ell ({\left(G^{r_{c}}\right)}^{2j+1}))\approx 0,k>0,j\geq 0$.

Proof.

As $G^{r_{c}}={\left(G^{r_{c}}\right)}^{1}$ is a chordal graph, then so are the graphs ${\left(G^{r_{c}}\right)}^{2j+1},j\geq 1$ (lemma 4.5). The fact that the homology of the clique complexes of these graphs is trivial follows from lemma 4.3. ▪

Thus, every entry corresponding to $C\ell ({\left(G^{r_{c}}\right)}^{2j+1}),j\geq 0$, in the associated β(G) binary barcode is 0, i.e. these 0 entries repeat in β(G) with a period of 2.

As indicated by lemma 4.6, it can be the case that $G^{2}$ is not chordal even though *G* is (however, if $G^{2}$ is chordal, then all powers $1\leq r<r_{s}$ of *G* are necessarily chordal [20] and *G* exhibits persistent triviality). This situation can produce transient ‘topological noise’ that is a feature peculiar to *r* filtrations and is described by the following corollary. Because this result is an obvious consequence of the last theorem, it is stated without proof.

**Corollary 5.6.**

*Assume that G is a chordal graph and* $G^{2}$ *is not a chordal graph. Then every non-zero homology class* $[c]\in H_{k}(C\ell (G^{2j})),k>0,j\geq 1$, *is transient noise*.

A special case of this—*persistent periodic transient noise*—arises when every $C\ell (G^{2j}),j\geq 1$, has at least one non-zero homology class in some non-zero dimension. In this case, the entries in the binary barcode β(G) corresponding to $C\ell (G^{2j}),j\geq 1$, are 1 (i.e. 1 repeats with period 2) and those corresponding to $C\ell (G^{2j+1}),j\geq 1$, are 0 (i.e. 0 repeats with period 2). Thus, beginning with the first location which corresponds to Cℓ(G) the pattern 01 is repeated for the remainder of the string (of course, the *r_s_*th and final entry in β(G) is 0). Note that even if $H_{k}(C\ell (G^{2}))\approx 0,k>0$, the fact that $G^{2}$ is not chordal signals a structural change in the relationships between subsets of vertices of *G* that may provide useful insights into the properties of *G* (e.g. see the sunflower graph example in the next section).

It is interesting to note (lemmas 4.4 and 4.6) the significance of sunflower subgraphs $S_{n}$ in determining the persistence and periodicity of homological triviality in *r* filtrations. Obviously, if $S_{n}⊈G$ and *G* is chordal, then *G* is closed under powers, the clique complexes of all powers of *G* (through its stabilization distance) are homologically trivial, and *G* exhibits persistent triviality. Perhaps not so obvious is the case where a sunflower subgraph in a chordal graph *G* prevents $G^{2}$ from being chordal. However, with a little reflection, it is easily seen that consecutive pairs of the independent vertices $\left\{u_{1},u_{2},\ldots ,u_{n}\right\}$ for each $S_{n}\subseteq G$ are separated by a graph distance of two and are therefore adjacent in $G^{2}$. This forms chordless cycles $C_{n}$ in $G^{2}$, thereby rendering it non-chordal and producing non-zero homology classes in $H_{1}(C\ell (G^{2}))$. Clearly, when $S_{n},n\geq 4$, is suspended in *G*, then *G* cannot be chordal because the suspension itself induces chordless cycles in *G*. Consequently, Cℓ(G) exhibits a non-trivial homology because these cycles generate non-zero homology classes in $H_{1}(C\ell (G))$.

## 6. Examples

It is worthwhile to illustrate several of the theorems developed above using graphs of relatively small order. As a first example consider the cycle graph $C_{11}$ and note that there is a counting number l=3>2 such that $3l+1=3\cdot 3+1=10<g(C_{11})=11$. As the cycle $C_{11}$ clearly induces the non-zero homology class $[c]\in H_{1}(C\ell (C_{11}))\approx Z$, then—from theorem 5.3—it must be the case that λ(c)≥3. In order to verify this, note that λ(c)<5 because $\mathrm{diam}(C_{11})=5=r_{s}(C_{11})$ so that $C_{11}^{5}≅K_{11}$ and $H_{1}(C\ell (C_{11}^{5}))\approx 0$. When r=2(3){4} then every three (four) {five} consecutive vertices in $C_{11}$ forms eleven 2(3){4}—simplices in $C\ell (C_{11}^{r})$—each of which contracts to a point and yields $C\ell (C_{11})$ as the resulting complex. Consequently, $H_{1}(C\ell (C_{11}))\approx H_{1}(C\ell (C_{11}^{2}))\approx H_{1}(C\ell (C_{11}^{3}))\approx H_{1}(C\ell (C_{11}^{4}))\approx Z$ and λ(c)=4≥3, thereby confirming the assertion of theorem 5.3. The associated binary *r* persistence barcode is the string $\beta (C_{11})=11110$.

This example also illustrates theorem 5.4. In particular, as $H_{1}(C\ell (C_{11}^{4}))\approx Z$, then $C_{11}^{4}$ is not chordal (via contrapositive of lemma 4.3 because $C_{11}^{4}$ is connected). It follows that $r_{c}(C_{11})=r_{s}(C_{11})=5$ and that $C_{11}^{r_{c}}=C_{11}^{5}≅K_{11}$ is a sunflower free chordal graph. This—when combined with the discussion in the last paragraph—validates theorem 5.4 because $\lambda (c)=4=r_{s}(C_{11})-1$.

**Figure 1.**
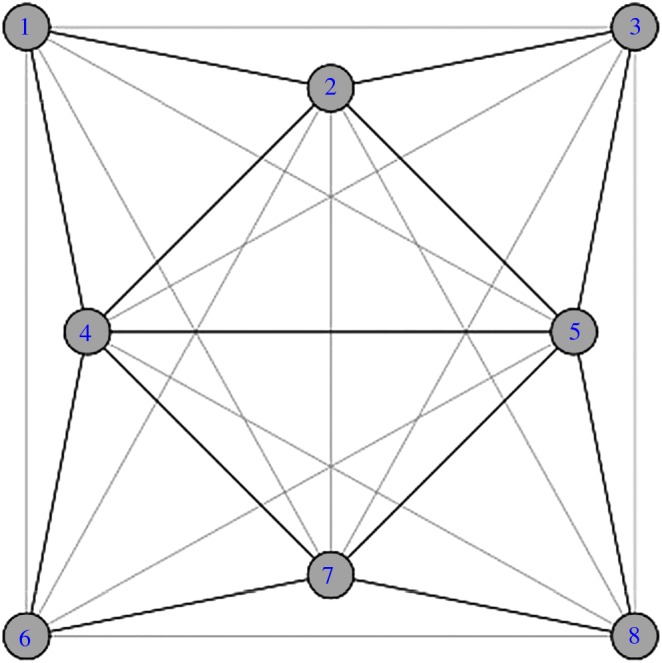
$S_{4}$ (dark edges) and $S_{4}^{2}$ (dark and light edges).

Now consider the sunflower graph $S_{4}$ (dark edges) and $S_{4}^{2}$ (dark and light edges) shown in figure 1. Although $S_{4}$ is chordal so that $r_{c}(S_{4})=1$, $S_{4}^{2}$ is not because the four cycle c=(1683) does not have a chord. However, $C\ell (S_{4}^{2})$ is homologically trivial as *c* can be contracted through the four 2—simplices corresponding to the petals of the sunflower onto the central square defined by the vertices 2,4,5 and 7. In addition, it is easily determined by inspection that $\mathrm{diam}(S_{4})=3=r_{S}(S_{4})$ so that $S_{4}^{3}≅K_{8}$. Thus, $H_{1}(C\ell (S_{4}))\approx H_{1}(C\ell (S_{4}^{2}))\approx H_{1}(C\ell (S_{4}^{3}))\approx 0$ and $S_{4}$ exhibits persistent triviality. The associated persistence binary barcode is $\beta (S_{4})=000$.

Because there are no non-zero homology classes in $H_{1}(C\ell (S_{4}^{2}))$ and therefore no transient noise, these results are consistent with corollary 5.6. Also—as per the discussion in the last section—the fact that $S_{4}$ is chordal and $S_{4}^{2}$ is not chordal could indicate potentially useful insights into the properties of $S_{4}$ (e.g. although vertices 1,6,8 and 3 are unrelated in $S_{4}$, the vertices in the vertex pairs 13,38,68 and 16 are close enough in $S_{4}$ to be related by a single unit change in *r*).

Both Rips filtrations and Čech filtrations are used in the analysis of large datasets in $R^{n}$. Recall that the Čech complex associated with a finite collection *Σ* of data points in $R^{n}$ is the abstract simplicial complex whose *k*-simplices are those subsets of k+1 data points in *Σ* whose closed ball neighbourhoods of radius *β* have a common point of intersection, whereas points in the simplices in the associated Rips complex are pairwise within a distance *ϵ*. A Čech (Rips) filtration of *Σ* is performed by varying the value of the ball radius *β* (the value of *ϵ*) and constructing a Čech (Rips) complex for each *β* (*ϵ*) value of interest.

In what follows, a Čech filtration of a dataset *Σ* of 30 points in $R^{n}$ is used to construct a series of graphs for Čech complex representations of *Σ*. For the purpose of comparison, an *r* filtration of an associated relative neighbourhood graph R representation of *Σ* is used to highlight several advantages that can be obtained by using power filtrations instead of Euclidean filtrations in the analysis of large datasets.

**Figure 2.**
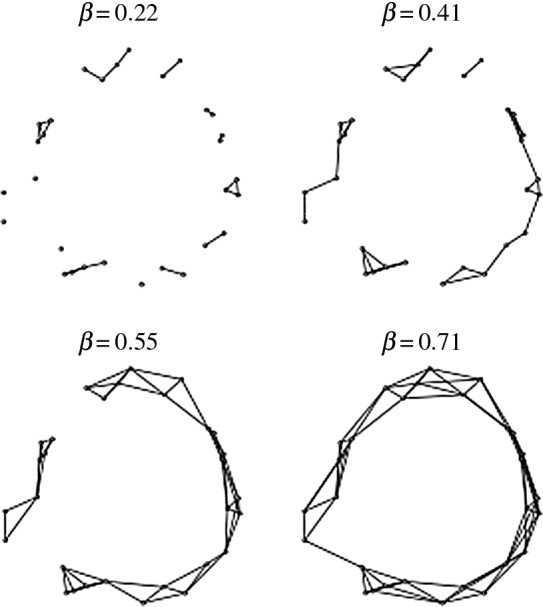
Graphs of Čech complexes obtained from a Čech filtration of dataset *Σ*.

The graphs of four Čech complexes obtained from a Čech filtration of the 30 point dataset *Σ* are shown in figure 2 for ball radius values β=0.22,0.41,0.55 and 0.71. As can be seen in the figure, the disconnected components do not persist: the graph of the initial complex ${\overset{ˇ}{C}}_{0.22}$ at β=0.22 is highly disconnected and the distribution of connected components of the graph of ${\overset{ˇ}{C}}_{0.22}$ in the figure is ‘circular’. As *β* increases, the number of connected components in the graphs of the associated complexes decreases until the graph of the complex ${\overset{ˇ}{C}}_{0.71}$ is completely connected around a ‘large one-dimensional hole’ *γ* at β=0.71. Although the homology group sequence $H_{0}({\overset{ˇ}{C}}_{0.22})\approx Z⊕Z⊕\cdots ⊕Z$ (14 copies of Z), $H_{0}({\overset{ˇ}{C}}_{0.41})\approx Z⊕Z⊕Z⊕Z⊕Z$, $H_{0}({\overset{ˇ}{C}}_{0.55})\approx Z⊕Z$ and $H_{0}({\overset{ˇ}{C}}_{0.71})\approx Z$ of the four complexes cannot explicitly detect *γ*, the lack of persistence in zero dimensional homology and the *β* range over which the rank of the zero-dimensional homology groups decreases from 14 to 1 provides a good description of the Euclidean compactness of *Σ* in $R^{n}$ (the presence of *γ* would be detected by $H_{1}({\overset{ˇ}{C}}_{0.71})$).

A relative neighbourhood graph [24] on a dataset *X* has *X* as its vertex set with an edge between $x_{i},x_{j}\in X$ if and only if $X\cap B(x_{i},d_{ij})\cap B(x_{j},d_{ij})=\emptyset$. Here, B(x,ρ) is the open ball in $R^{n}$ of radius *ρ* and centre *x*, and $d_{ij}=d(x_{i},x_{j})$ is the Euclidean distance between $x_{i}$ and $x_{j}$. The importance of a relative neighbourhood graph of *X* is that it provides a single graph representation of *X* that serves as a ‘primal sketch’ of its topological features.

**Figure 3.**
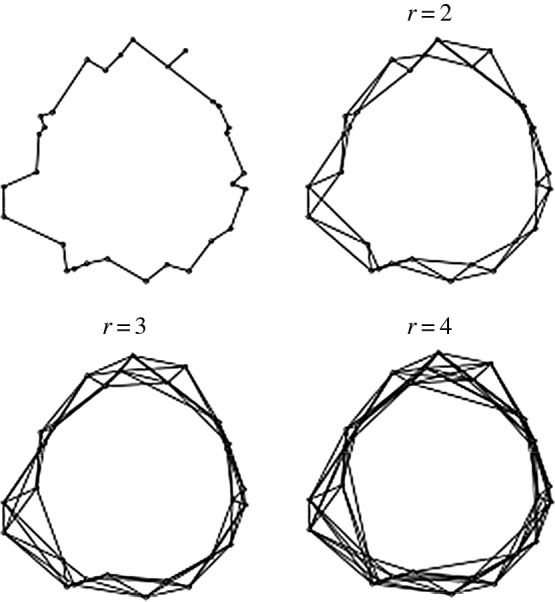
The relative neighbourhood graph R and its first three powers for dataset *Σ*.

The relative neighbourhood graph R and its first three powers for the 30 element dataset *Σ* are shown in figure 3. Observe that: (i) R immediately exhibits the connected cyclic structure of *Σ* that eventually emerged in the Čech filtration of *Σ* at a sufficiently large *β* value; and (ii) the cyclic structure of *Σ* persists in $R^{r},1\leq r\leq 4$. Because Cℓ(R) is connected and if *c* is the cycle in R so that $0\neq [c]\in H_{1}(C\ell (R))$, then—by inspection—$H_{0}(C\ell (R))\approx Z\approx H_{1}(C\ell (R))$ and $H_{k}(C\ell (R))\approx 0,k>1$. However, more can be deduced about the persistence of homological features in $C\ell (R^{r})$ by first noting that g(R)=29 and then by applying lemma 4.1, theorems 5.2 and 5.3. In particular, when l=9>2, then 3l+1=28<29=g(R) in which case $H_{k}(C\ell (R))\approx H_{k}(C\ell (R^{2}))\approx \cdots \approx H_{k}(C\ell (R^{9})),k\geq 0.$ Thus, $H_{0}(C\ell (R^{r}))\approx Z,1\leq r\leq r_{s}(R)$, $H_{1}(C\ell (R^{r}))\approx Z,1\leq r\leq 9$ so that λ(c)≥9, and $H_{k}(C\ell (R^{r}))\approx 0,1\leq r\leq 9,k>1$.

**Figure 4.**
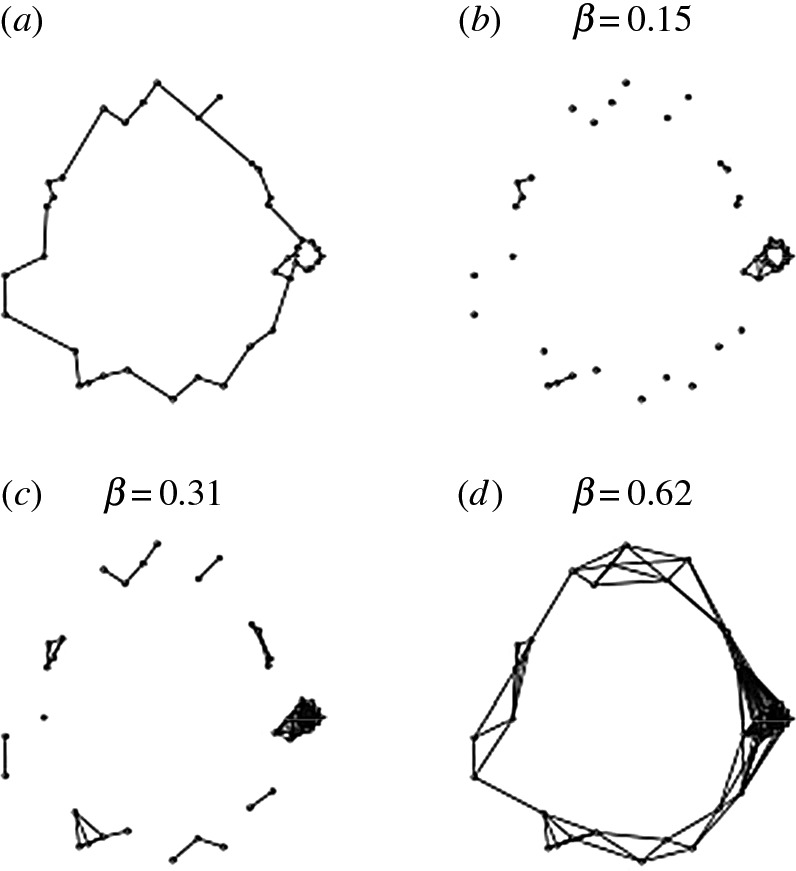
(*a*–*d*) Comparison of *Γ* with graphs of Čech complexes obtained from a Čech filtration of *Ψ*.

As a final example, consider the relative neighbourhood graph *Γ* representation of another dataset Ψ≠Σ consisting of 40 data points in $R^{n}$ shown in figure 4*a*. Observe that the single graph *Γ* immediately discerns three cycles (at different scales), whereas they only gradually emerge from a Čech filtration of *Ψ* as *β* increases. The power filtration of *Γ* provides essentially the same information about *Ψ* as the Čech filtration but—unlike the Čech filtration—it has the advantage that (in this case) it provides this information with a single graphical representation of *Ψ* so that *Ψ* is accessed only once to compute *Γ*. It is easy to see from theorem 5.2 that $H_{0}(C\ell (\Gamma^{r}))\approx Z,1\leq r\leq r_{s}$. However, observe that as g(Γ)=5<10, theorem 5.3 cannot be applied to the filtration of *Γ*.

## 7. Closing remarks

This paper has introduced the notion of using homological persistence in simplicial complexes obtained from power filtrations of simple graphs as an approach to probing their topological structure. This method is especially useful when applied to graphs with girths greater than 10. In these cases, the homologies of complexes in the filtration remain isomorphic and persist over a range of power indices that increases with increasing girth. An interesting feature of power filtrations of graphs is the fact that the emergence of a pre-stabilization chordal graph in a filtration signals the presence of trivial or periodic homology which persists for the remainder of the filtration.

Using as examples datasets in $R^{n}$, it is also suggested that: (i) for those cases where a data-derived graph is designed to elicit information of interest and the graph can be computed efficiently, power filtration provides an alternative approach to persistent homology analysis while providing information comparable to that obtained from Čech or Rips filtrations and (ii) because it requires only an initial single graph representation of a dataset, power filtrations tend to reduce ‘topological noise’ in many practical applications.

In closing, it is important to note that the results of this paper can be applied directly to such cases as physical network and social network analysis where the data are naturally represented as a simple graph. In addition, in manifold learning a single simple graph is constructed and used to produce a data embedding into a lower dimensional space. The results of this paper provide a mechanism for understanding the topology of this graph along with the potential for producing additional information relevant to the associated embedding [25].

## Data accessibility

The following R code was used to generate the example 30 point dataset *Σ* in §6:

set.seed(435243)
N <- 30
theta <- seq(0,2*pi,length=N+1)[-1]
z <- cbind(cos(theta)+rnorm(N,0,.1),sin(theta)+rnorm(N,0,.1))

The following R code was used to append dataset *Σ* with 10 additional points to produce the example 40 point dataset *Ψ* in §6:

set.seed(89820)
r <- .1
n <- 10
thetar <- seq(0,2*pi,length=n+1)[-1]
x <- cbind(r*cos(thetar)+rnorm(n,0,.1*r),r*(sin(thetar)+rnorm(n,0,.1*r))
y <- rbind(z,x)
